# Evaluation of Corneal Irregular Astigmatism and Visual Quality Following Bilateral Sequential SMILE and LASEK: A Six‐Year Comparative Study

**DOI:** 10.1155/joph/5989651

**Published:** 2026-02-08

**Authors:** Hua Li, Weinan Hu, Min Li, Yuehui Shi, Lina Sun, Xiaoyun Ma, Jun Zou

**Affiliations:** ^1^ Department of Ophthalmology, Shanghai Tenth People’s Hospital, School of Medicine, Tongji University, Shanghai, China, tongji.edu.cn; ^2^ Department of Ophthalmology, Zhoupu Hospital, Shanghai University of Medicine and Health Sciences, Shanghai, China, sumhs.edu.cn

**Keywords:** Fourier analysis, irregular astigmatism, LASEK, SMILE, visual quality

## Abstract

**Purpose:**

To compare the corneal spherical component (SC), regular astigmatism (RA), irregular astigmatism (IA, including asymmetry and irregularity), and visual quality 6 years after small incision lenticule extraction (SMILE) and laser subepithelial keratomileusis (LASEK) for mild‐to‐moderate myopia.

**Methods:**

This retrospective, comparative study comprised the SMILE group (35 eyes) and LASEK group (36 eyes). Visual acuity, corneal topography utilizing swept‐source anterior segment OCT, and wavefront aberrations were recorded preoperatively and 6 years postoperatively. Fourier analysis of keratometric‐derived parameters of the anterior, posterior, and total cornea at 6 mm zone was evaluated.

**Results:**

Six years postoperatively, the safety and efficacy indices were comparable between both groups. Fourier analysis revealed significant changes in SC decrease and asymmetry increase of the anterior and total cornea (*p* < 0.001), with LASEK exhibiting a more pronounced flattening effect of the anterior cornea (*p* = 0.001). Interestingly, RA of the anterior and total cornea decreased significantly after LASEK (*p* = 0.016,  0.002, respectively). Further linear correlation analysis showed that changes in SC (Δ SC) of anterior cornea and total cornea were correlated with the preoperative spherical power, mean refractive spherical equivalent (MRSE), lenticule thickness/ablation depth, ΔK1, and ΔK2 (all *|r|* > 0.85, *p* < 0.001). Compared with LASEK, SMILE induced less horizontal coma at 6 years postoperatively (*p* = 0.008).

**Conclusions:**

Both SMILE and LASEK are safe and effective procedures for correction of mild‐to‐moderate myopia. LASEK demonstrates an advantage in flattening the anterior cornea and reducing regular astigmatism, while SMILE exhibits superior performance in inducing less horizontal coma

**Trial Registration:** ClinicalTrials.gov identifier: NCT06673992

## 1. Introduction

Laser lamellar corneal refractive surgery, such as small incision lenticule extraction (SMILE), and laser surface corneal refractive surgery like laser subepithelial keratomileusis (LASEK) have emerged as highly effective, safe, predictable, and stable procedures for correcting mild‐to‐moderate myopia [1]. Both SMILE and LASEK are flapless refractive techniques that avoid complications related to corneal biomechanics damage and induction of higher‐order aberrations (HOAs) due to the creation of a corneal flap. Some studies have suggested that the biomechanical advantages after SMILE were comparable to those of LASEK [2]. In terms of visual quality, previous studies have shown that SMILE induced fewer HOAs compared with LASEK at 3 months postoperatively [3] and less corneal spherical aberration but greater vertical coma than LASEK at 2 years postoperatively [1, 4]. However, the underlying reasons for these differences remain poorly understood.

As is well known, alterations in corneal morphology are the primary contributors to refractive system aberrations, which predominantly impact postoperative visual quality. Previous studies have investigated the corneal changes induced by SMILE, FS‐LASIK, and LASEK techniques [2, 5–9]. Additionally, Fourier analysis has demonstrated its ability to detect subtle changes in corneal shape caused by corneal disorders or surgery [10–12]. Fourier analysis enables separate quantification of the spherical component (SC), regular astigmatism (RA), and asymmetry and irregularity of the cornea. SC and RA values are directly proportional to keratometric spherical and astigmatic powers. Asymmetry refers to a tilt of the cornea with respect to the videokeratoscope axis while irregularity reflects a series of optical imperfections that degrade retinal image quality [13, 14]. The induced asymmetry and irregularity cannot be corrected using a spherocylindrical lens and thus represent overall corneal irregular astigmatism (IA) in a broad sense [15]. Moreover, it has been proposed that IA increased after PRK, T‐PRK, LASIK, FS‐LASIK, and SMILE procedures significantly impacting postoperative visual quality [14, 16–19]. Regarding the evaluation methods of IA, some studies have compared IA with Placido‐based topographer and three‐dimensional anterior segment optical coherence tomography (AS‐OCT) in dry eye, which proved that the advent of AS‐OCT can accurately quantify corneal configuration of the anterior and posterior corneal surface.

To the best of our knowledge, there is currently no existing study that compares corneal IA and visual quality following SMILE and LASEK for the correction of mild‐to‐moderate myopia at long‐term follow‐up. The primary objective of this study is to compare postoperative corneal IA after SMILE and LASEK using the AS‐OCT imaging technique and evaluate its impact on visual quality.

## 2. Patients/Materials and Methods

### 2.1. Patients

The Ethical Board Committee at Shanghai Tenth People’s Hospital, Tongji University, approved this study protocol (22K226), which adhered to the tenets of the Declaration of Helsinki. Informed consent for surgery was obtained from all patients.

This was a retrospective study performed at Shanghai Tenth People’s Hospital, and patients with mild‐to‐moderate myopia or myopic astigmatism who underwent SMILE or LASEK between October 2017 and May 2018 were enrolled. Inclusion criteria were as follows: (1) patients aged over 18 years; (2) absence of corneal, ocular, or systemic diseases; (3) stable refractive diopter (D) maintained for the past 2 years with a change of ± 0.50 D; (4) discontinuation of soft contact lens wear for at least 2 weeks and rigid contact lenses for at least 4 weeks prior to surgery; and (5) preoperative manifest refraction spherical equivalent (MRSE) less than −6.00 D. Out of a total of 71 eyes, SMILE was performed on 35 eyes of 20 patients, while LASEK was performed on 36 eyes of 18 patients.

### 2.2. Measurements

All patients underwent a comprehensive ophthalmic evaluation which included autorefraction, pupillometry, uncorrected distance visual acuity (UDVA), corrected distance visual acuity (CDVA), manifest and cycloplegic refraction, slit‐lamp examination of the cornea, anterior segment, and fundus, intraocular pressure (NCT, TX‐F, Canon, Japan) measurement, axial length (AL, IOL Master 700, Germany) assessment, and corneal tomography performed with CASIA SS1000 (Tomey Corporation, Nagoya, Aichi‐ken, Japan). Wavefront aberration metrics were evaluated within a 6‐mm zone utilizing Zyoptix Wavefront Aberration System (Zywave, Bausch& Lomb, USA). All indicators were measured 3 times repeatedly, and the average values were taken. The patients were reevaluated after 6 years on a voluntary basis. The 6‐year follow‐up examination included the evaluation of UDVA and CDVA, corneal tomography, wavefront aberration, and slit‐lamp examination.

### 2.3. Corneal Topography Measurements

The swept‐source AS‐OCT (CASIA SS‐1000) was utilized to acquire corneal topography measurements. In the Corneal Map mode, 16 radial cross‐sectional images (512 telecentric + A‐scans) were obtained within a duration of 0.3 s for each measurement. The analysis program identified and digitized the anterior and posterior corneal surfaces to evaluate distributions of corneal power and corneal height for both the anterior and posterior corneal surfaces. All subjects were examined at least twice to obtain well‐focused, properly‐aligned images of the eye. Using Fourier harmonic analysis program, the dioptric power of the ring *i*, *F*_*i*_ (*σ*), was transformed into trigonometric components of the following formula [13, 14]:

(1) Fiσ=α0+c1cos123σ−α1+c2cosσ−α2+c3cosσ−α3+⋯+cncosnσ−αn,

where *α*_0_ represents the SC of the ring, 2 × *c*_1_ represents corneal asymmetry, 2 × *c*_2_ represents RA, and the summation of *c*_3_, …, *c*_*n*_ includes the irregularity components. These indices were calculated within the central 6 mm diameter zone.

### 2.4. Wavefront Aberration Measurements

The wavefront aberrations under mesopic condition were measured and analyzed for 6‐mm pupil diameter with a Zywave aberrometer using a Hartmann–Shack sensor (Bausch & Lomb, US). The absolute Zernike coefficients including vertical trefoil, horizontal trefoil, vertical coma, horizontal coma, spherical aberration, and total higher‐order aberrations (tHOAs) were examined.

### 2.5. Surgical Procedures

The surgical procedures were all performed by the same surgeon (J.Z.). Following routine balanced salt solution (BSS) irrigation of the conjunctival sac and periocular sterilization, topical anesthesia was administered using two to three drops of procaine hydrochloride (Alcon, Inc., Geneva, Switzerland) twice prior to surgery.

#### 2.5.1. SMILE Procedure

The SMILE procedure was exclusively performed using the femtosecond laser platform (VisuMax; Carl Zeiss Meditec AG, Jena, Germany), with a typical pulse energy of approximately 150 nJ and a pulse repetition rate of 500 kHz. Four sequential cleavages were created to generate an intrastromal lenticule with the side cutting of a 3‐mm width incision at the 12:00 location of the cornea. The diameter of the lenticule was 6.7 mm while maintaining a corneal cap thickness of 120 μm. Additionally, a basement layer ranging from 10 to 20 μm beneath the lenticule was included to ensure successful removal.

#### 2.5.2. LASEK Procedure

In the LASEK group, an 8.5‐mm‐diameter corneal epithelial flap was created using a 20% alcohol for a duration of 12–15 s and subsequently removed with a crescent‐shaped corneal spatula, leaving a hinge at the 12 o’clock position. Corneal stromal tissue ablation was performed utilizing a MEL 90 excimer laser (Carl Zeiss Meditec, Jena, Germany) at a repetition rate of 500 Hz. The epithelial flap was repositioned, and a corneal bandage contact lens (ACUVUE OASYS; Johnson & Johnson, USA) was worn for 7 days.

Patients of SMILE group were administered with topical 0.5% levofloxacin (Cravit; Santen, Inc.) eye drops four times daily for 7 days and 0.1% fluorometholone (FML; Flumetholon; Santen, Inc.) four times daily for 2 weeks and then gradually reduced to once every 2 weeks. Patients of LASEK group were treated with topical 0.5% levofloxacin eye drops four times daily for 7 days and 0.1% FML four times daily for 1 month followed by tapering down to once every month.

### 2.6. Statistical Analysis

The statistical software package SPSS (Version 20.0, SPSS, Inc., Armonk, NY, USA) was utilized for conducting descriptive statistics. Continuous variables were presented as means ± standard deviations. The *Shapiro–Wilk* test was employed to assess the normality of the data distribution. Gender comparison between the two groups was conducted using the chi‐square test. Mixed‐effects models with Bonferroni‐adjusted post hoc comparisons were applied for analyzing the Fourier analysis parameters and ocular wavefront aberrations parameters between SMILE and LASEK which can eliminate intereye correlations of both eyes. Generalized estimating equations (GEEs) were used to evaluate the variation of Fourier analysis parameters (Δ = postop‐preop) between SMILE and LASEK. The Spearman correlations with Bonferroni correction were conducted to explore the relationships between Fourier analysis parameters and influencing factors before and after both surgeries (a total of 76 comparisons). A Bonferroni‐adjusted *p* value of < 0.00066 (0.05/76) was used for correlation analysis significance.

## 3. Results

### 3.1. Demographic Characteristics of Patients

The preoperative baseline characteristics of patients by group are presented in Table 1. At the 6‐year follow‐up, postoperative examination was completed for 35 eyes in the SMILE and 36 eyes in the LASEK groups. There were no significant differences observed in the preoperative baseline characteristics between the two groups. However, a significant difference was found in ablation depth (AD)/lenticule thickness (LT) and optical zone between the two groups as shown in Table 1.

**TABLE 1:** Preoperative demographic and refractive data for both groups.

Parameter	SMILE group	LASEK group	*χ*/*t* value	*p* value
Sex (male/female)	2/18	6/12	3.103	0.078
*N* (eyes)	35	36		
Age (year)	26.88 ± 5.51	29.65 ± 6.00	1.451	0.156
Spherical (D)	−3.63 ± 1.07	−4.03 ± 1.13	−1.525	0.132
Cylinder (D)	−0.44 ± 0.47	−0.63 ± 0.48	−1.663	0.080
MRSE (D)	−3.85 ± 1.08	−4.34 ± 1.13	−1.887	0.063
CCT (μm)	533.80 ± 26.06	520.44 ± 38.23	−1.715	0.091
Axial length (mm)	25.37 ± 0.89	25.26 ± 0.72	−0.579	0.564
Vertical coma	0.19 ± 0.21	0.12 ± 0.09	−1.870	0.066
Horizontal coma	0.09 ± 0.10	0.06 ± 0.14	−1.175	0.244
Vertical trefoil	0.18 ± 0.15	0.12 ± 0.08	−1.969	0.053
Horizontal trefoil	0.11 ± 0.08	0.10 ± 0.07	−0.885	0.379
Spherical aberrations	0.36 ± 0.23	0.30 ± 0.10	−1.385	0.170
tHOAs	6.36 ± 1.73	6.78 ± 1.52	1.074	0.287
Optical zone (mm)	6.70 ± 0.00	6.51 ± 0.15	−7.519	0.000
LT/AD (μm)	92.54 ± 18.25	79.36 ± 15.62	−3.273	0.002

*Note:* SMILE = small incision lenticule extraction, LASEK = laser subepithelial keratomileusis.

Abbreviations: AD = ablation depth, CCT = central corneal thickness, LT = lenticule thickness, MRSE = mean refractive spherical equivalent, SD = standard deviation, and tHOAs = total higher‐order aberrations.

### 3.2. Visual Outcomes

The 6‐year efficacy index (postoperative UDVA/preoperative CDVA) in the SMILE and LASEK groups was 0.97 ± 0.08 and 1.00 ± 0.11, respectively (*p* = 0.322). 100% (35/35) of SMILE‐treated eyes (Figure 1(a)) and 97% (35/36) of LASEK‐treated eyes (Figure 2(a)) had a postoperative UDVA of 0 or better (log MAR). Additionally, 100% (35/35) of SMILE‐treated eyes (Figure 1(a)) and 97% (35/36) of LASEK‐treated eyes (Figure 2(a)) had a UDVA equal to or better than preoperative CDVA. Furthermore, 97% (34/35) of SMILE‐treated eyes (Figure 1(d)) and 95% (34/36) of LASEK‐treated eyes (Figure 2(d)) had postoperative MRSE within ±0.50D, and all the eyes had postoperative MRSE within ±1.00D.

FIGURE 1Refractive outcomes of 35 patients with mild‐to‐moderate myopia after SMILE 6 years postoperatively. UDVA = uncorrected distance visual acuity, CDVA = corrected distance visual acuity, Postop = postoperative, Preop = preoperative, and D = diopter.

**Figure:**
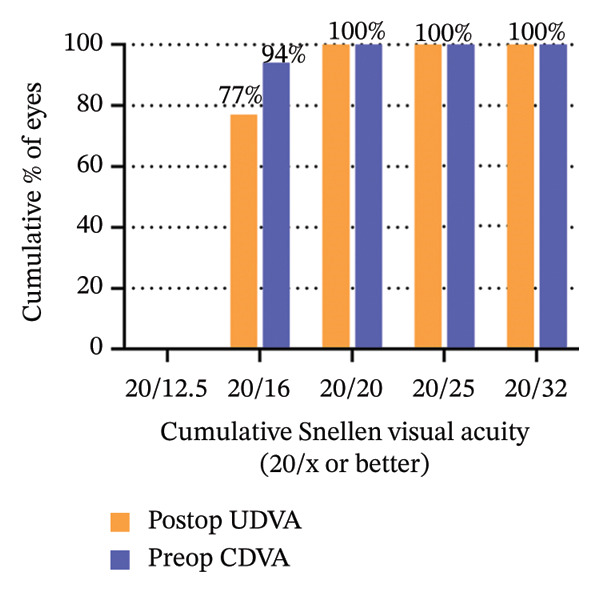
(a)

**Figure:**
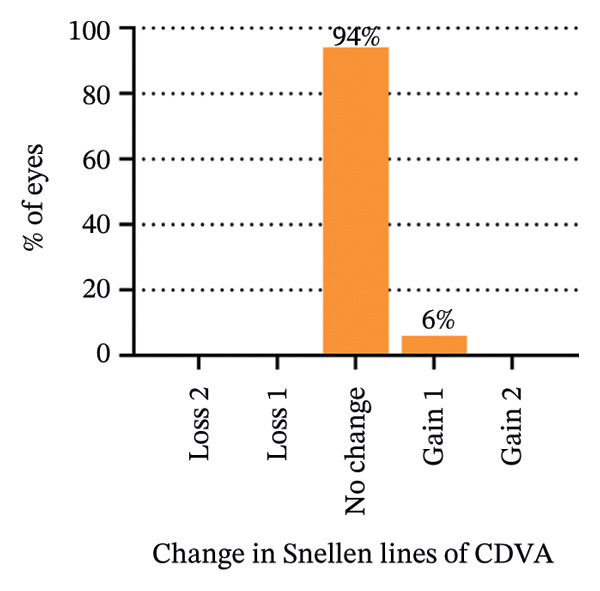
(b)

**Figure:**
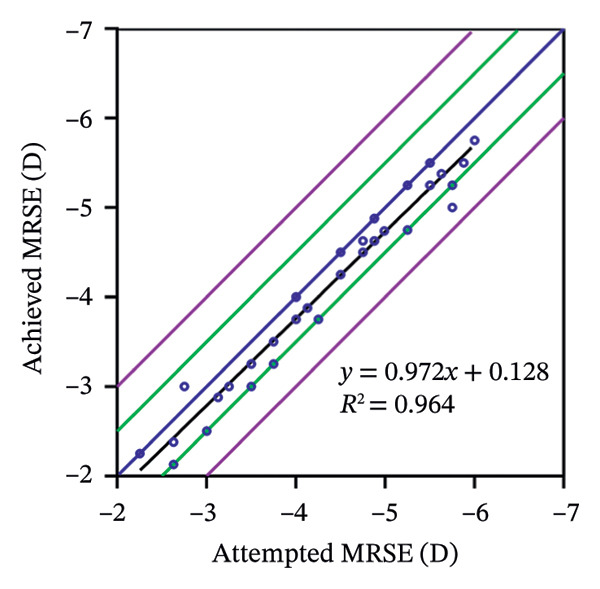
(c)

**Figure:**
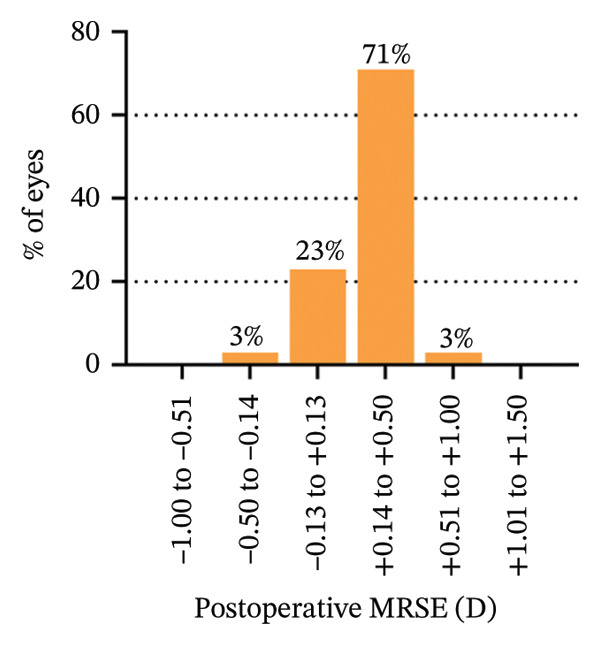
(d)

**Figure:**
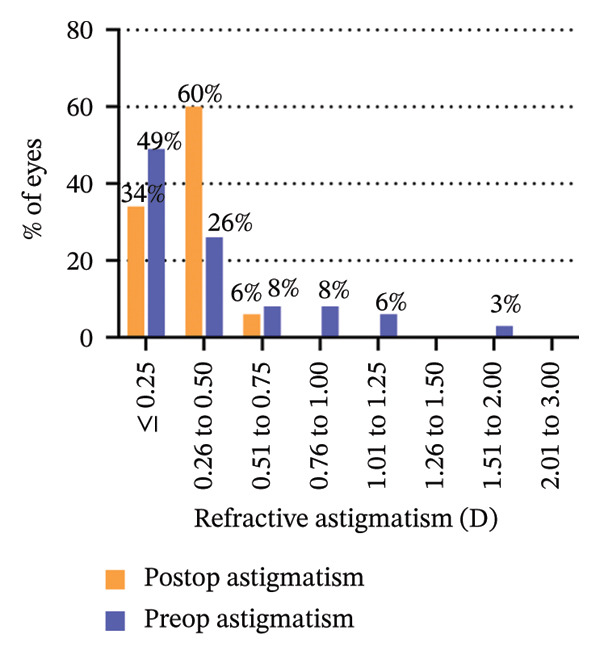
(e)

FIGURE 2Refractive outcomes of 36 patients with mild‐to‐moderate myopia after LASEK 6 years postoperatively. UDVA = uncorrected distance visual acuity, CDVA = corrected distance visual acuity, Postop = postoperative, Preop = preoperative, and D = diopter.

**Figure:**
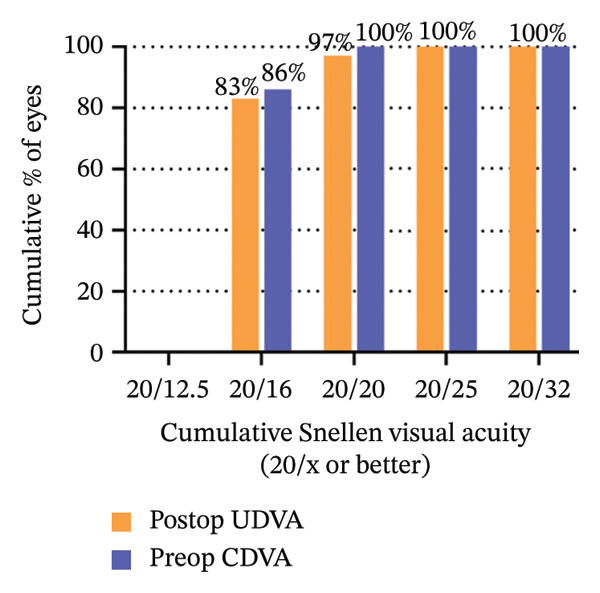
(a)

**Figure:**
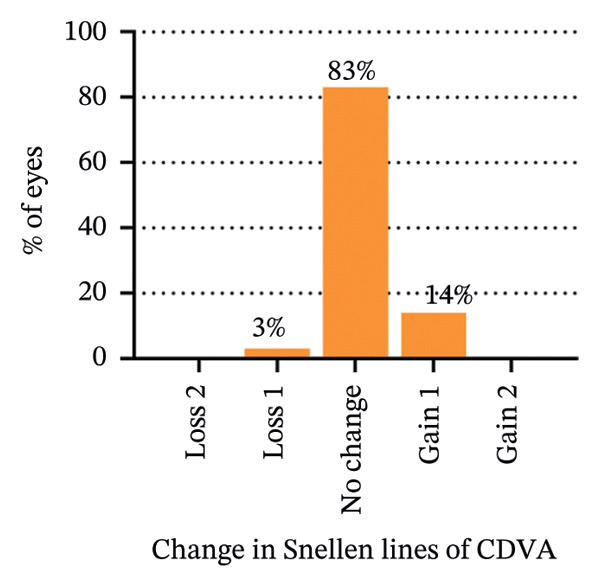
(b)

**Figure:**
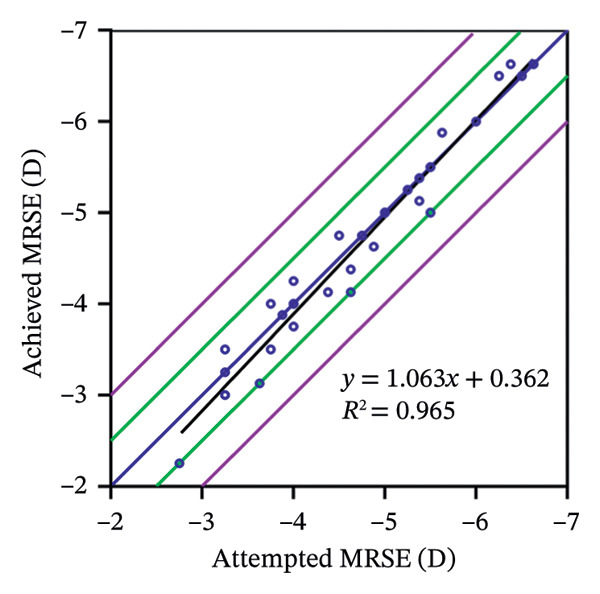
(c)

**Figure:**
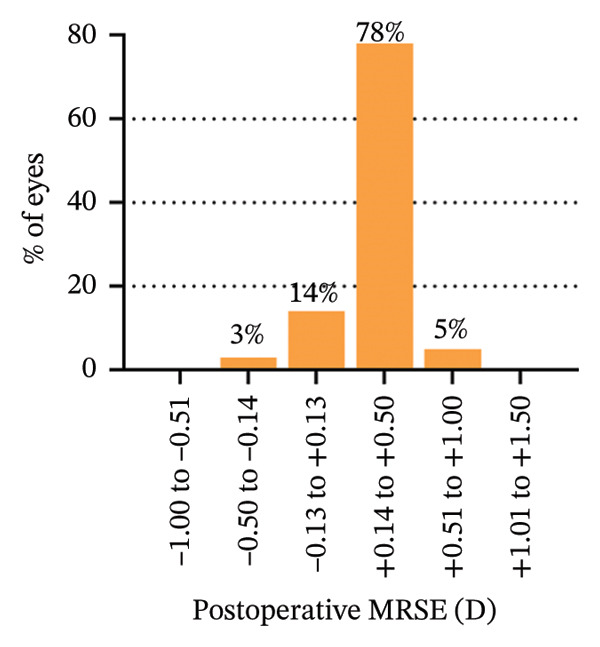
(d)

**Figure:**
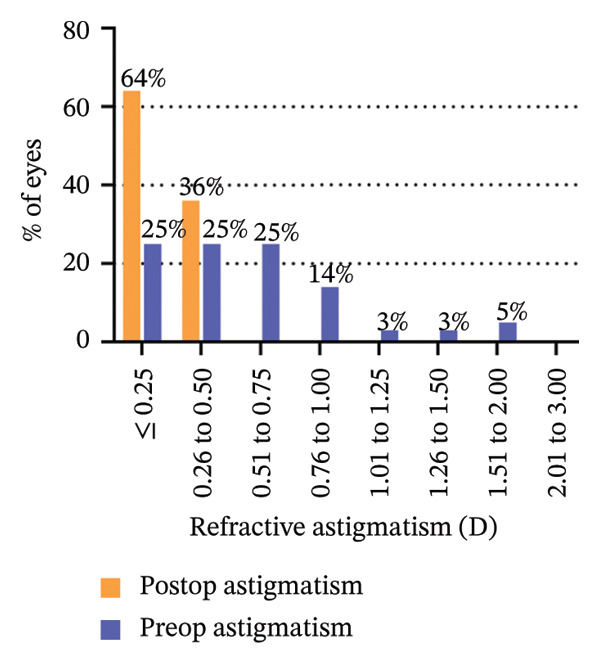
(e)

The safety index (postoperative CDVA/preoperative CDVA) of the SMILE group and LASEK group was 1.01 ± 0.05 and 1.02 ± 0.08, respectively (*p* = 0.449). 6% (2/35) of SMILE‐treated eyes (Figure 1(b)) and 14% (5/35) of LASEK‐treated eyes (Figure 2(b)) gained a one‐line improvement in CDVA. In either group, no eyes lost two or more lines of CDVA.

The predictability is demonstrated through scatter plots depicting the disparity between the target spherical equivalent (SE) and the achieved SE of the SMILE and LASEK groups, respectively (Figures 1(c) and 2(c)).

In terms of stability, the postoperative SE values for the SMILE and LASEK groups were 0.25 ± 0.20D and 0.35 ± 0.23D, correspondingly. The postoperative astigmatism in the SMILE (Figure 1(e)) and LASEK (Figure 2(e)) groups was −0.34 ± 0.26D and −0.18 ± 0.24D, respectively.

### 3.3. Fourier Analysis Parameters

#### 3.3.1. Anterior Cornea

The changes in Fourier analysis parameters after SMILE and LASEK are summarized in Table 2. Following SMILE and LASEK, there was a significant decrease in mean SC over the anterior cornea within the 6‐mm zone (*p* < 0.001), while asymmetry showed a significant increase (*p* < 0.001). In addition, RA significantly decreased after LASEK (*F* = 5.954, *p* = 0.016), but there was no significant difference in this value after SMILE. A comparison of induced changes in Fourier analysis parameters between the two groups revealed that LASEK resulted in more change of anterior cornea SC and RA compared to SMILE (χ^2^ = 10.209, *p* = 0.001; χ^2^ = 8.026, *p* = 0.005, respectively).

**TABLE 2:** The comparison of Fourier analysis parameters after SMILE and LASEK^a^.

Variables	Mean ± SD		*F* ^b^	*p* ^b^	*F* ^c^	*p* ^c^
	Preoperative	Postoperative				
*Anterior cornea SC*						
SMILE group	47.85 ± 0.27	44.50 ± 0.27	79.769	< 0.001	0.336	0.546
LASEK group	48.45 ± 0.26	44.28 ± 0.26	127.545	< 0.001		

*Anterior cornea RA*						
SMILE group	0.44 ± 0.04	0.46 ± 0.04	0.186	0.667	0.843	0.360
LASEK group	0.63 ± 0.04	0.51 ± 0.04	5.954	0.016		

*Anterior cornea Asymmetry*						
SMILE group	0.40 ± 0.05	0.88 ± 0.05	41.870	< 0.001	6.410	0.012
LASEK group	0.30 ± 0.05	0.69 ± 0.05	29.088	< 0.001		

*Anterior cornea irregularity*						
SMILE group	0.16 ± 0.01	0.15 ± 0.01	0.021	0.886	1.249	0.266
LASEK group	0.16 ± 0.01	0.16 ± 0.01	0.116	0.734		

*Posterior cornea SC*						
SMILE group	−6.05 ± 0.04	−6.08 ± 0.04	0.211	0.647	2.021	0.157
LASEK group	−6.15 ± 0.04	−6.15 ± 0.04	0.001	0.971		

*Posterior cornea RA*						
SMILE group	0.12 ± 0.01	0.13 ± 0.01	0.295	0.588	14.853	< 0.001
LASEK group	0.16 ± 0.01	0.17 ± 0.01	1.260	0.264		

*Posterior cornea* asymmetry						
SMILE group	0.06 ± 0.00	0.06 ± 0.00	0.003	0.958	3.176	0.077
LASEK group	0.04 ± 0.00	0.05 ± 0.00	0.526	0.470		

*Posterior cornea irregularity*						
SMILE group	0.02 ± 0.00	0.02 ± 0.00	0.569	0.452	1.489	0.224
LASEK group	0.02 ± 0.00	0.02 ± 0.00	5.547	0.020		

*Total cornea SC*						
SMILE group	41.92 ± 0.24	38.52 ± 0.24	102.900	< 0.001	0.829	0.364
LASEK group	42.42 ± 0.23	38.22 ± 0.23	161.627	< 0.001		

*Total cornea RA*						
SMILE group	0.34 ± 0.03	0.35 ± 0.03	0.115	0.736	0.104	0.748
LASEK group	0.50 ± 0.03	0.37 ± 0.03	10.289	0.002		

*Total cornea asymmetry*						
SMILE group	0.38 ± 0.05	0.86 ± 0.05	43.920	< 0.001	5.430	0.021
LASEK group	0.29 ± 0.05	0.69 ± 0.05	30.883	< 0.001		

*Total cornea irregularity*						
SMILE group	0.15 ± 0.01	0.15 ± 0.01	0.013	0.910	0.902	0.344
LASEK group	0.16 ± 0.01	0.16 ± 0.01	0.013	0.911		

*Note:* SMILE = small incision lenticule extraction, LASEK = laser subepithelial keratomileusis.

Abbreviation: SD = standard deviation.

^a^Total eyes = 71 (SMILE group: *n* = 35; LASEK group: *n* = 36).

^b^ *p* values represent the comparison between preoperative and postoperative of both surgeries, in which < 0.05 were considered significant when mixed‐effects models with Bonferroni‐adjusted post hoc comparisons were performed.

^c^ *p* values represent the comparison of postoperative values between SMILE and LASEK, in which < 0.05 were considered significant when mixed‐effects models with Bonferroni‐adjusted post hoc comparisons were performed.

#### 3.3.2. Posterior Cornea

Following LASEK, there were no significant alterations observed in the posterior SC, RA, and asymmetry (*p* > 0.05), while there was a significant increase in posterior corneal irregularities (*F* = 5.547, *p* = 0.020, refer to Table 2). The SMILE procedure did not result in any statistically significant changes in the posterior cornea across all examined variables (*p* > 0.05).

#### 3.3.3. Total Cornea

After SMILE and LASEK, the mean SC over the 6‐mm zone total cornea exhibited a significant decrease (*p* < 0.001), while the asymmetry showed a significant increase (*p* < 0.001). In addition, the total cornea RA significantly decreased after LASEK (*F* = 10.289, *p* = 0.002), but there was no significant difference in this value after SMILE. When comparing changes in these parameters between the two groups on the total cornea, it was observed that LASEK induced a greater increase in total cornea SC and RA compared with the SMILE group (χ^2^ = 9.426, *p* = 0.002; χ^2^ = 9.086, *p* = 0.003, respectively).

### 3.4. Ocular Wavefront Aberrations

The changes of ocular wavefront aberrations after SMILE and LASEK are presented in Table 3. Postoperatively, both groups exhibited an increase in vertical coma, horizontal coma, spherical aberration, and tHOAs (all *p* < 0.005). A comparison of the postoperative ocular wavefront aberrations between the two groups revealed that LASEK resulted in a greater horizontal coma at 6 years postoperatively (*p* = 0.008), while SMILE resulted in a greater vertical coma (*p* = 0.027).

**TABLE 3:** The comparison of ocular high order aberrations following SMILE and LASEK^a^.

Variables	Mean ± SD		*F* ^b^	*p* ^b^	*F* ^c^	*p* ^c^
	Preoperative	Postoperative				
*Vertical trefoil*						
SMILE group	0.18 ± 0.02	0.15 ± 0.02	0.631	0.428	0.181	0.671
LASEK group	0.12 ± 0.02	0.14 ± 0.02	0.283	0.596		

*Horizontal trefoil*						
SMILE group	0.11 ± 0.02	0.11 ± 0.02	0.002	0.969	0.542	0.463
LASEK group	0.10 ± 0.07	0.13 ± 0.02	2.080	0.151		

*Vertical coma*						
SMILE group	0.19 ± 0.03	0.35 ± 0.03	13.899	< 0.001	4.973	0.027
LASEK group	0.12 ± 0.03	0.25 ± 0.03	9.889	0.002		

*Horizontal coma*						
SMILE group	0.09 ± 0.03	0.24 ± 0.03	16.041	< 0.001	7.211	0.008
LASEK group	0.11 ± 0.03	0.34 ± 0.03	38.679	< 0.001		

*Spherical aberrations*						
SMILE group	0.36 ± 0.04	0.54 ± 0.04	14.177	< 0.001	0.069	0.793
LASEK group	0.30 ± 0.04	0.56 ± 0.04	27.872	< 0.001		

*tHOAs*						
SMILE group	0.42 ± 0.04	0.64 ± 0.04	18.259	< 0.001	1.181	0.279
LASEK group	0.36 ± 0.04	0.70 ± 0.04	41.668	< 0.001		

*Note:* SMILE = small incision lenticule extraction; LASEK = laser subepithelial keratomileusis.

Abbreviations: SD = standard deviation; tHOAs = total higher‐order aberrations.

^a^Total eyes = 71 (SMILE group: *n* = 35; LASEK group: *n* = 36).

^b^ *p* values represent the comparison between preoperative and postoperative of both surgeries, in which < 0.05 were considered significant when mixed‐effects models with Bonferroni‐adjusted post hoc comparisons were performed.

^c^ *p* values represent the comparison of postoperative values between SMILE and LASEK, in which < 0.05 were considered significant when mixed‐effects models with Bonferroni‐adjusted post hoc comparisons were performed.

### 3.5. Linear Correlation Analysis

**FIGURE 3:**
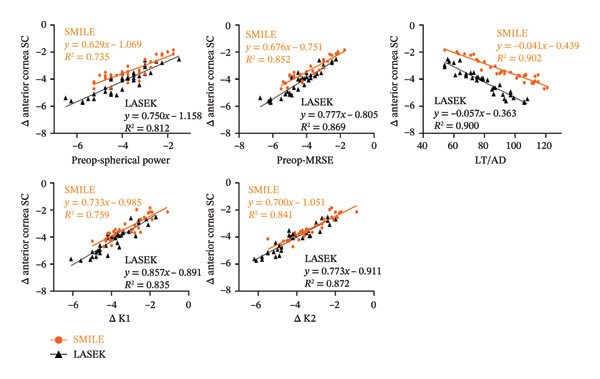
Alterations in anterior cornea SC plotted as a function of changes in preoperative spherical power, preoperative MRSE, LT/AD, Δ K1, and Δ K2.

**FIGURE 4:**
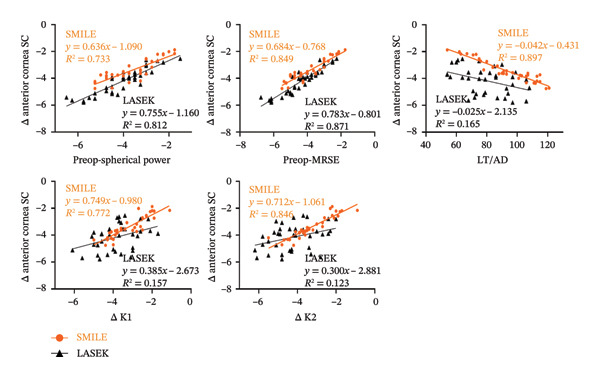
Alterations in total cornea SC plotted as a function of changes in preoperative spherical power, preoperative MRSE, LT/AD, Δ K1, and Δ K2.

The results of the correlation analysis for changes in Fourier analysis parameters in the SMILE group are presented in Table 4. Significant correlations were observed between changes in SC of anterior cornea (Δ anterior cornea SC) and total cornea (Δ total cornea SC) with preoperative spherical power, MRSE, LT, ΔK1, and ΔK2 (Figures 3 and 4).

**TABLE 4:** Correlation analysis of variations in Fourier analysis parameters and influencing factors before and after SMILE surgery^a^.

Variable	Δ anterior cornea SC	Δ anterior cornea asymmetry	Δ total cornea SC	Δ total cornea asymmetry
	*r* *p*	*r* *p*	*r* *p*	*r* *p*
Preop‐spherical power	0.857, 0.000^∗^	−0.070, 0.690	0.856, 0.000^∗^	−0.068, 0.698
Preop‐cylinder	0.305, 0.075	−0.147, 0.400	0.304, 0.076	−0.135, 0.439
Preop‐MRSE	0.923, 0.000^∗^	−0.102, 0.558	0.921, 0.000^∗^	−0.098, 0.575
Preop‐K1	−0.346, 0.042	−0.429, 0.010	−0.354, 0.037	−0.419, 0.012
Preop‐K2	−0.439, 0.008	−0.353, 0.037	−0.443, 0.008	−0.347, 0.041
LT/AD	−0.950, 0.000^∗^	0.097, 0.578	−0.947, 0.000^∗^	0.095, 0.588
Postop‐spherical power	−0.072, 0.681	0.255, 0.139	−0.079, 0.651	0.265, 0.124
Postop‐cylinder	0.302, 0.078	−0.464, 0.005	0.307, 0.073	−0.451, 0.007
Postop‐MRSE	0.109, 0.533	−0.003, 0.988	0.104, 0.554	0.017, 0.924
Postop‐K1	0.242, 0.161	−0.437, 0.009	0.238, 0.168	−0.418, 0.012
Δ K1	0.871, 0.000^∗^	0.082, 0.641	0.878, 0.000^∗^	0.090, 0.608
Postop‐K2	0.225, 0.195	−0.391, 0.020	0.222, 0.200	−0.376, 0.026
Δ K2	0.917, 0.000^∗^	0.047, 0.788	0.920, 0.000^∗^	0.056, 0.750
Postop‐vertical trefoil	0.068, 0.698	−0.528, 0.001	0.073, 0.678	−0.532, 0.001
Postop‐horizontal trefoil	−0.388, 0.021	−0.117, 0.505	−0.395, 0.019	−0.129, 0.461
Postop‐vertical coma	−0.078, 0.656	0.388, 0.021	−0.071, 0.687	0.416, 0.013
Postop‐horizontal coma	−0.169, 0.331	0.026, 0.884	−0.164, 0.345	0.026, 0.881
Postop‐spherical aberrations	−0.186, 0.285	0.050, 0.775	−0.178, 0.305	0.069, 0.694
Postop‐tHOAs	−0.128, 0.465	−0.043, 0.808	−0.118, 0.499	−0.042, 0.812

*Note:* Δ = postoperative value‐preoperative value.

Abbreviation: tHOAs = total higher‐order aberrations.

^a^Total eyes of SMILE group = 35.

^∗^A Bonferroni‐adjusted value of < 0.00066 (0.05/76) was used for correlation analysis significance.

The correlation analysis results of the Fourier analysis parameters in the LASEK group are presented in Table 5. The changes in SC of the anterior cornea and total cornea were significantly correlated with preoperative spherical power, MRSE, AD, ΔK1, ΔK2, and postoperative K1 and K2 (Figures 3 and 4).

**Table 5:** Correlation analysis of variations in Fourier analysis parameters and influencing factors before and after LASEK surgery^a^.

Variable	Δ anterior cornea SC	Δ anterior cornea asymmetry	Δ total cornea SC	Δ total cornea asymmetry
	*r* *p*	*r* *p*	*r* *p*	*r* *p*
Preop‐spherical power	0.901, 0.000^∗^	−0.279, 0.100	0.901, 0.000^∗^	−0.296, 0.080
Preop‐cylinder	0.134, 0.434	−0.292, 0.084	0.138, 0.422	−0.284, 0.093
Preop‐MRSE	0.932, 0.000^∗^	−0.342, 0.041	0.933, 0.000^∗^	−0.358, 0.032
Preop‐K1	0.137, 0.424	−0.023, 0.894	0.138, 0.422	−0.006, 0.972
Preop‐K2	0.043, 0.804	0.084, 0.625	0.042, 0.809	0.109, 0.526
LT/AD	−0.949, 0.000^∗^	0.297, 0.079	−0.951, 0.000^∗^	0.308, 0.068
Postop‐spherical power	−0.180, 0.294	0.085, 0.622	−0.177, 0.302	0.102, 0.552
Postop‐cylinder	0.237, 0.164	−0.133, 0.440	0.240, 0.159	−0.142, 0.410
Postop‐MRSE	−0.051, 0.769	0.013, 0.941	−0.047, 0.788	0.025, 0.883
Postop‐K1	0.627, 0.000^∗^	−0.126, 0.464	0.628, 0.000^∗^	−0.125, 0.467
Δ K1	0.914, 0.000^∗^	−0.190, 0.268	0.915, 0.000^∗^	−0.211, 0.218
Postop‐K2	0.639, 0.000^∗^	−0.139, 0.417	0.640, 0.000^∗^	−0.132, 0.442
Δ K2	0.934, 0.000^∗^	−0.324, 0.054	0.936, 0.000^∗^	−0.345, 0.039
Postop‐vertical trefoil	0.223, 0.191	−0.100, 0.562	0.222, 0.194	−0.107, 0.536
Postop‐horizontal trefoil	−0.072, 0.676	−0.066, 0.700	−0.076, 0.660	−0.064, 0.712
Postop‐vertical coma	0.057, 0.741	0.320, 0.057	0.055, 0.751	0.316, 0.060
Postop‐horizontal coma	−0.125, 0.469	0.313, 0.063	−0.123, 0.475	0.317, 0.060
Postop‐spherical aberrations	−0.072, 0.676	0.344, 0.040	−0.074, 0.670	0.343, 0.040
Postop‐tHOAs	0.033, 0.849	0.148, 0.389	0.037, 0.831	0.152, 0.376

*Note:* Δ = postoperative value‐preoperative value.

Abbreviation: tHOAs = total higher‐order aberrations.

^a^Total eyes of LASEK group = 36.

^∗^A Bonferroni‐adjusted value of < 0.00066 (0.05/76) was used for correlation analysis significance.

## 4. Discussion

In the current study, we decomposed the corneal topography data of advanced swept‐source AS‐OCT (CASIA SS‐1000) into a series of trigonometric functions using Fourier harmonic analysis and quantified the amount of asymmetry and irregularity components. We successfully compared postoperative IA after SMILE and LASEK and evaluated its effects on visual quality over a 6‐year follow‐up. Additionally, we compared the basic refractive effect and visual quality after SMILE and LASEK.

The postoperative visual outcome is a primary concern for both patients and doctors. Our study demonstrated that there was no significant difference in the efficacy and safety index between SMILE and LASEK at the 6‐year follow‐up, indicating their comparable safety and effectiveness in treating mild‐to‐moderate myopia. These findings were consistent with those of Yu et al. [3], Cai et al. [20], and Liu et al. [21], who reported no difference in visual outcomes after SMILE and LASEK. Therefore, we can choose either appropriate surgical protocol for patients with different cornea thickness and refractive conditions, as their long‐term visual outcomes were consistent.

Regarding the Fourier analysis of corneal topography, previous research has reported significant increases in asymmetry and irregularity of the anterior corneal surface after both FS‐LASIK and SMILE surgeries. Additionally, both procedures have shown a significant flattening effect [14]. Consistent with these results, our study demonstrated that 6 years after SMILE and LASEK, there was a significant decrease in SC and an increase in asymmetry of the anterior and total cornea within the 6‐mm zone. Notably, LASEK resulted in a more pronounced flattening effect on the anterior cornea within this region compared to SMILE. This difference could be attributed to the distinct ablation principles employed by each technique. During SMILE surgery, a “cap” is created which includes the corneal epithelium, the Bowman’s layer, and part of the anterior stroma. Myopia correction involves creating and removing a lenticule beneath this “cap.” Due to its presence, there might be a potential gap between the “cap” and the residual corneal stroma. The flattening effect observed with SMILE depends on intraocular pressure, the “cap,” as well as overall corneal biomechanics [22, 23]. In contrast, for LASEK, the surface stroma was ablated directly with excimer laser after cornea epithelium removed. Therefore, LASEK treatments seem more direct and effective means of altering the anterior surface of the cornea. Furthermore, the optical zone for LASEK (6.51 ± 0.15 mm) is smaller than SMILE (6.70 mm), and this difference may affect the comparisons of corneal flattening and corneal asymmetry. Wang et al. [24] reported that the asphericity of the cornea was positively correlated with the effective optical zone. For a given D correction, the optical zone design directly influences the AD, which may affect postoperative corneal epithelial remodeling and morphology. However, this aspect requires further investigation. Surprisingly, the RA of the anterior and total cornea decreased significantly after LASEK; however, in SMILE, the RA only showed an increasing trend but without statistical significance. This evidence was in accordance with previous studies, which suggested that SMILE surgically induced lower astigmatism, and LASEK exhibited the advantage of reducing corneal astigmatism. Similarly, the refractive results indicate that the proportions of astigmatism within 0.25D after SMILE and LASEK surgeries are 34% and 64%, respectively. This discrepancy may also be attributed to the distinct ablation principles of the two techniques. In astigmatism correction during refractive surgery, the application of the excimer laser in LASEK is more direct and effective compared to the femtosecond laser used in SMILE.

In addition to correcting refractive error for optimal vision, achieving perfect and natural visual quality after surgery is the ultimate objective for both surgeons and patients. This is because the induction of HOAs can lead to significant night vision issues such as glare, haze, and halos. In previous comparative studies, Zhu et al. found that SMILE has lower HOAs and spherical aberration induction rate than LASEK for high myopia correction 1 year postoperatively [25]. Similarly, Yu et al. revealed that SMILE induced less HOAs, especially spherical aberration, than LASEK at 3 months postoperatively [3]. Fu et al. suggested that SMILE induced less corneal spherical aberration but greater vertical coma than LASEK at 2 years postoperatively [1]. This finding was also observed by Cai et al., who studied the corneal aberration by Pentacam, showing lower HOAs, spherical aberration, and horizontal comas of the anterior and whole corneal surfaces were observed in the SMILE group than in the LASEK group at 3 months and 1 year postoperatively. However, the vertical coma of the anterior surface and total cornea in the SMILE group was significantly greater than that in the LASEK group [20]. Similarly, we found that the total ocular vertical coma, horizontal coma, spherical aberration, and tHOAs were increased after both surgeries. SMILE induced less total ocular horizontal coma and greater vertical coma than LASEK at 6 years postoperatively. This phenomenon may be attributed to the following factors: (i) As demonstrated in our study, SMILE surgery can lead to increased corneal asymmetry, with correlation analysis further indicating an association between vertical coma and corneal asymmetry (Table 4); (ii) the SMILE incision was located above the cornea, and the postoperative healing response causes an upper and lower cornea asymmetry, resulting in a relatively larger vertical coma; and (iii) there was no eye movement tracking system in SMILE operation, and eccentric ablation may cause the increase of high‐order aberrations, especially the increase of coma [26].

Regarding the factors influencing the Fourier analysis parameters of the cornea topography, we conducted a series of related analyses. We discovered significant correlations between changes in SC of the anterior cornea and total cornea as well as various preoperative factors including spherical power, MRSE, LT/AD, ΔK1, and ΔK2. These findings suggested that the changes in SC were associated with the corneal removal profile, which may explain why there was a difference in SC between the two procedures. These findings were consistent with those of Ning and Zhang who reported that the postoperative SC correlated with the depth of corneal tissue removal and the optical zone after SMILE and t‐PRK [17]. This discrepancy of related factors may be associated with the different refractive surgeries between both studies.

## 5. Conclusion

The current 6‐year retrospective study indicates that both SMILE and LASEK are excellent surgical options for the correction of mild‐to‐moderate myopia. LASEK demonstrates an advantage in flattening the anterior cornea and reducing regular astigmatism, while SMILE exhibits superior performance in inducing less horizontal coma.

## 6. Limitations

A limitation of our study is the inclusion of both eyes with relatively small sample; we have used the mixed‐effects models to account for within‐patient intereye correlations. Another limitation is the lack of an analysis of the optical zone on corneal Fourier parameters, which should be a focus of future research.

## Author Contributions

Hua Li and Weinan Hu are co‐first authors.

## Funding

This research was supported by the Clinical Research Project of Shanghai Municipal Health Commission (202240131), the Program for Research‐Oriented Physician of Shanghai Tenth People’s Hospital (YJXYS‐B‐009), the Clinical Research Center of Shanghai Tenth People’s Hospital (YNCR2C004), the Clinical Research Project of Community Eye Health Management and Evaluation of the Effects of Integrated Traditional Chinese and Western Medicine Therapy (2023SQ03), and the National Natural Science Foundation of China (82301162).

## Conflicts of Interest

The authors declare no conflicts of interest.

## Data Availability

The data that support the findings of this study are available from the corresponding authors upon reasonable request.
